# Antioxidant, Antimicrobial Effects and Phenolic Profile of *Lycium barbarum* L. Flowers

**DOI:** 10.3390/molecules200815060

**Published:** 2015-08-17

**Authors:** Andrei Mocan, Laurian Vlase, Dan Cristian Vodnar, Ana-Maria Gheldiu, Radu Oprean, Gianina Crișan

**Affiliations:** 1 Department of Pharmaceutical Botany, Iuliu Hațieganu University of Medicine and Pharmacy, 23 Ghe. Marinescu Street, Cluj-Napoca 400010, Romania; E-Mails: mocan.andrei@umfcluj.ro (A.M.); gcrisan@umfcluj.ro (G.C.); 2 Department of Pharmaceutical Technology and Biopharmaceutics, Iuliu Hațieganu University of Medicine and Pharmacy, 12 I. Creangă Street, Cluj-Napoca 400010, Romania; E-Mail: gheldiu.ana@umfcluj.ro; 3 Department of Food Science, University of Agricultural Sciences and Veterinary Medicine, 3-5 Manăştur Street, Cluj-Napoca 400372, Romania; E-Mail: dan.vodnar@usamvcluj.ro; 4 Department of Analytical Chemistry and Instrumental Analysis, Iuliu Hațieganu University of Medicine and Pharmacy, 4 L. Pasteur Street, Cluj-Napoca 400010, Romania; E-Mail: roprean@umfcluj.ro

**Keywords:** antioxidants, antimicrobial, polyphenols, *Lycium barbarum* L., flowers, EPR spectroscopy

## Abstract

*L. barbarum* L. is a widely-accepted nutraceutical presenting highly advantageous nutritive and antioxidant properties. Its flowers have been previously described as a source of diosgenin, β-sitosterol and lanosterol that can be further pharmaceutically developed, but no other data regarding their composition is available. The purpose of this work was to investigate the chemical constituents, antioxidant and antimicrobial activities of *L. barbarum* flowers, as an alternative resource of naturally-occurring antioxidant compounds. The free radical scavenging activity of the ethanolic extract was tested by TEAC, two enzymatic assays with more physiological relevance and EPR spectroscopy. The presence of several phenolic compounds, such as chlorogenic, *p*-coumaric and ferulic acids, but also isoquercitrin, rutin and quercitrin, was assessed by an HPLC/MS method. The antioxidant assays revealed that the extract exhibited a moderate antioxidant potential. The antimicrobial activity was mild against Gram-positive bacteria and lacking against *Escherichia coli*. These findings complete the scarce existing data and offer new perspectives for further pharmaceutical valorization of *L. barbarum* flowers.

## 1. Introduction

Natural products have been and continue to be a source of inspiration for a substantial fraction of human therapeutics. The pharmaceutical arsenal is significantly indebted to Nature and in particular to natural products obtained from traditional medicinal plants, fungi and bacteria [1]. Plant-based drugs continue to play an essential role in healthcare, and their usage by different cultures has been extensively documented.

The Romanian flora comprises many natural resources that possess an important role in people’s lives and can be ultimately valued as medicinal plants [2]. The genus *Lycium* (*Solanaceae*) gathers approximately 70 representatives who vegetate in separate and distinct regions distributed from the temperate to the subtropical regions of Eurasia, North America, South America, southern Africa and Australia [3]. The Romanian flora includes only two representatives of the *Lycium* genus, *Lycium barbarum* L. and *Lycium chinense* Mill., and mentions the first as being spontaneous and the second as cultivated [4]. *L. barbarum* has become extremely popular in the last few years due to its public acceptance as a nutraceutical with highly advantageous nutritive and antioxidant properties. Its fruits have been extensively studied for their anti-aging, neuroprotective, anti-fatigue, hypoglycemic, increasing metabolism, anti-cancer, cytoprotective, immunomodulatory and antioxidant effects [5]. However, recently, other plant parts have gathered the attention of the scientific community. Thus, *Lycium* leaves have been previously described as a valuable source of antioxidant and antimicrobial compounds [6]. Data regarding the chemical composition of *L. barbarum* flowers is scarce. Only one previous report could be identified that describes *L. barbarum* flowers as a source of diosgenin, β-sitosterol and lanosterol that can be further pharmaceutically developed [7]. In this context, in the present study, the crude ethanolic extract of *L. barbarum* fresh flowers was evaluated towards its phenolic content by using several photometric assays and high-performance liquid chromatography coupled with mass spectrometry (HPLC/MS). Further, the antioxidant potential was tested through different electron-transfer assays, two enzymatic methods and EPR spectroscopy and the antimicrobial effect by a disk diffusion method.

## 2. Results and Discussion

### 2.1. HPLC/MS Analysis of Phenolic Compounds

**Figure 1:**
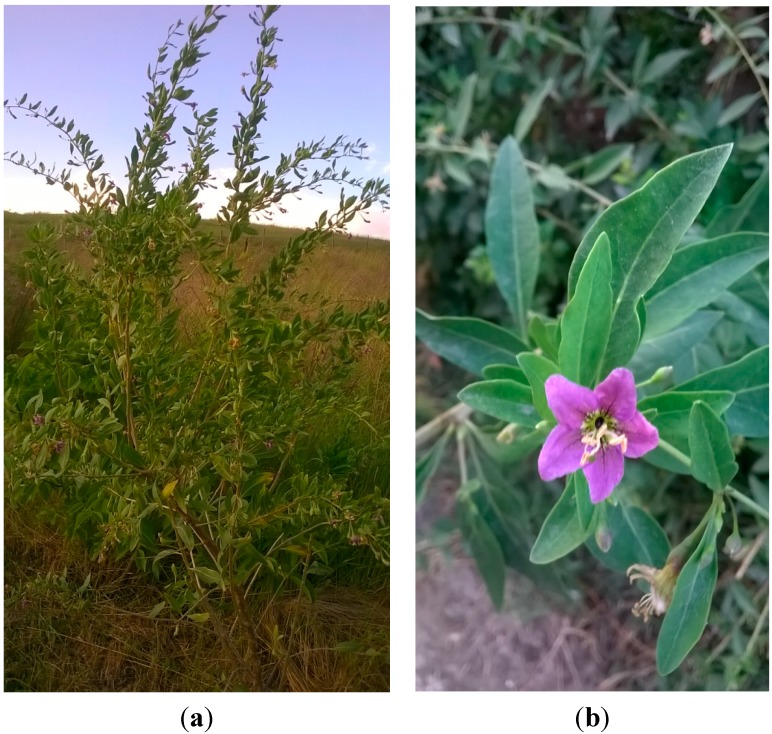
(**a**) General picture of *L. barbarum*; (**b**) detailed picture of the *L. barbarum* flower.

An HPLC/MS method was applied for the identification and quantification of phenolic acids, flavonoid glycosides and aglycones in the 70% (*v*/*v*) ethanolic extracts of *L. barbarum* flowers (Figure 1) [8,9].

In this study, a target analysis regarding eighteen standard phenolic compounds was used for the investigation of the ethanolic extracts. The method allows a simultaneous analysis of different classes of polyphenols by a single column pass, in 35 min [10]. Each sample was analyzed before and after acid hydrolysis in order to obtain more accurate data on flavonoid glycosides and aglycones concentration and to estimate the nature of hydrolyzed compounds [11]. The amounts of identified polyphenolic compounds in the analyzed samples are reported in Table 1.

**Table 1:** The polyphenolic compounds content in *Lycium barbarum* flower extracts (μg/g fresh matter).

Polyphenolic Compounds	*m*/*z* Value	R_T_ ± SD (min)	Extract	Hydrolyzed Extract
Caffeic acid	179	6.52 ± 0.04	-	˂0.2
Chlorogenic acid	353	6.43 ± 0.05	216.91 ± 2.19	˂0.2
*p*-Coumaric acid	163	9.48 ± 0.08	12.84 ± 0.03	6.21 ± 0.01
Ferulic acid	193	12.8 ± 0.10	42.48 ± 0.03	42.98 ± 0.52
Sinapic acid	223	15.00 ± 0.10	-	2.82 ± 0.02
Isoquercitrin	463	20.29 ± 0.10	20.46 ± 0.01	-
Rutin	609	20.76 ± 0.15	60.53 ± 0.04	-
Quercitrin	447	23.64 ± 0.13	11.13 ± 0.01	-
Quercetin	301	27.55 ± 0.15	-	75.51 ± 0.07
Kaempferol	285	32.48 ± 0.17	-	89.73 ± 0.05

-, Not found. Each value represents the mean ± SD (*n* = 3).

Nine compounds were identified in both extracts. Three phenolic acids (chlorogenic, *p*-coumaric and ferulic acids) and three quercetin glycosides (isoquercitrin, rutin and quercitrin) were observed prior to the hydrolysis. The dominant compound among the phenolic acids was chlorogenic acid (216.91 ± 2.19 μg/g f.w. (fresh weight)). Regarding the flavonoid pattern, rutin represented the major flavonoid (60.53 ± 0.04 μg/g f.w.). These results are in line with our previous findings considering *L. barbarum* leaves and suggest that chlorogenic acid and rutin are the major compounds in both natural products [6]. The presence of sinapic acid, quercetin (75.51 ± 0.07 μg/g f.w.) and kaempferol (89.73 ± 0.05 μg/g f.w.) in the hydrolyzed extract suggests the release of these compounds from their corresponding glycosylated structures.

### 2.2. Total Phenolic and Flavonoids Content

For comparative purposes, we also determined total phenolic and flavonoid content in the extract by using colorimetry with Folin-Ciocâlteu reagent and aluminium chloride, respectively. The total phenolic content (TPC) of the plant extract was expressed as milligrams of gallic acid equivalents per gram of fresh material (mg GAE/g f.w.) and the total flavonoids content as milligrams of quercetin equivalents per gram of fresh material (mg QE/g f.w.), the results being summarized in Table 2.

**Table 2:** Total content of phenolics (TPC) and flavonoids.

Total Bioactive Compounds	*Lycium barbarum* Flower Extract
TPC (mg/g fresh weight GAE)	3.75 ± 0.13
Total flavonoids (mg/g fresh weight QE)	0.61 ± 0.02

GAE: gallic acid equivalents; QE: quercetin equivalents; each value represents the mean ± SD (*n* = 3).

As witnessed by our previous findings concerning *L. barbarum* leaves, *L. barbarum* flowers contain lower amounts of total flavonoids and phenolic compounds compared to the leaves; however, our results regarding the leaves were expressed in the matter of dry weight vegetal material [6].

### 2.3. Free Radical Scavenging Activity

**Figure 2:**
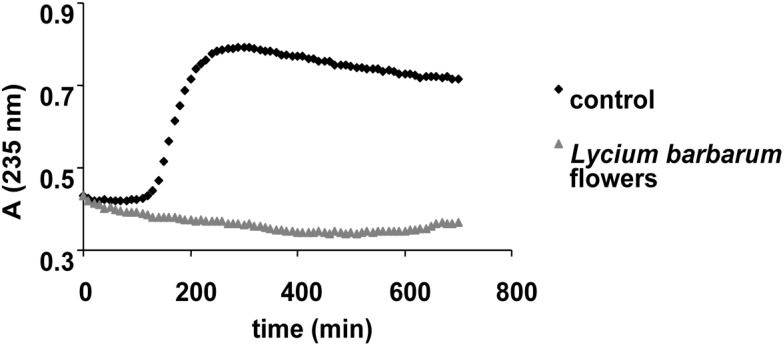
Liposome oxidation by cytochrome *c*, in the presence of the tested sample.

**Figure 3:**
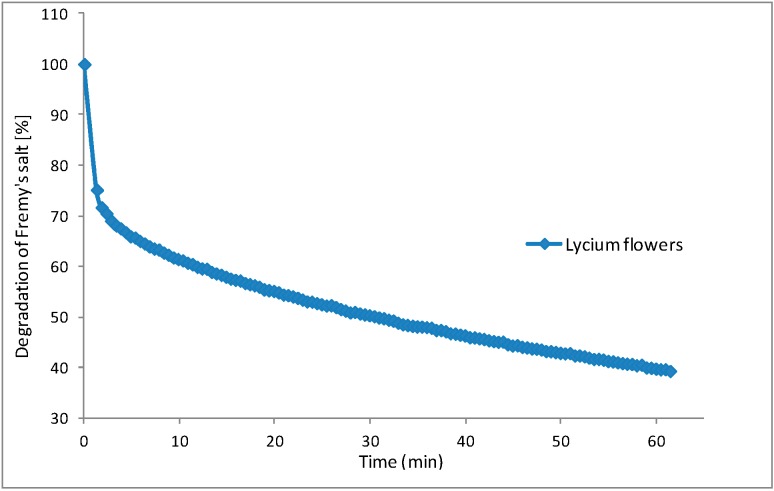
Degradation kinetics of the free radical Fremy’s salt by *L. barbarum* flowers extract.

The antioxidant activity of *Lycium barbarum* flower extract was assessed by Trolox equivalent antioxidant capacity (TEAC), the hemoglobin ascorbate peroxidase activity inhibition (HAPX) assay, EPR spectroscopy and by testing its effect on the inhibition of lipid peroxidation catalyzed by cytochrome *c*, as witnessed in Table 3 and Figure 2 and Figure 3. The radical scavenging capacity of the extract against the stable synthetic ABTS radical was expressed as Trolox equivalents (7.10 ± 0.28 mg TE/g f.w.).

**Table 3:** Antioxidant capacity parameters. TEAC, Trolox equivalent antioxidant capacity; HAPX, hemoglobin ascorbate peroxidase activity inhibition.

Samples	TEAC (mg TE/g f.w.)	HAPX (%)	EPR (mg FSE/g f.w.)
*L. barbarum* flowers	7.10 ± 0.28	9.45 ± 2.66	29.84 ± 2.56

TE: Trolox equivalents; Fremy’s salt equivalents. Each value represents the mean ± SD (*n* = 3).

The enzymatic antioxidant assay (HAPX) measures the ability of the extract bioactive compounds to quench the damage inflicted by hydrogen peroxide upon hemoglobin [12]. Additional information is provided by this assay, since it involves the interaction between antioxidant molecules with a protein, the ferryl hemoglobin species (resulting from the action of hydrogen peroxide on ferric hemoglobin). In this case, the *L. barbarum* flower extract presented a 9.45 ± 2.66 percent of inhibition. Similar results were obtained by Benedec *et al.* by testing extracts of *Achillea distans* subsp. *alpina* and lower on *A. distans* subsp. *distans* by means of the same method [13]. A more physiological method based on peroxidase activity of cytochrome *c* was also applied to evaluate the antioxidant activity of the *L. barbarum* flowers extract. This process monitors the formation of conjugated lipid dienes at 235 nm. Thus, the antioxidant capacity of the tested extract, reflected in the delay of lipid peroxidation, is considered to be based on the same mechanism found in HAPX: the interaction of antioxidants with ferryl hemoglobin, generated in this case by cytochrome *c* [14]. In this assay, the extract blocked the process of lipid peroxidation for the whole duration of the experiment (700 min), as can be observed in Figure 2.

The use of EPR applied to assessing antioxidant activity in foods or medicinal plants systems has become frequent due to its advantageous applications [15]. Antioxidants in *L. barbarum* flower crude extract were assessed by electron paramagnetic resonance (EPR) spectrometry to evaluate their efficiency to reduce a synthetic free radical species, *i.e.*, the semi-stable nitroxide radical Fremy’s salt (potassium nitrosodisulfonate) (Figure 3).

Information about antioxidant activity of *L. barbarum* flowers, using the EPR spectrometry assay, is not available, so far. This technique was used in the present study to support the results obtained by the traditionally-used TEAC and enzymatic assays. This might give additional information about the antioxidant capacity of different plant extracts investigated due to the fact that when investigating the antioxidant activity of a natural compound or extracts, more than one assay should be performed [16]. In this case, the investigated extract presented the ability to scavenge approximately 60.7% of free radicals (29.84 ± 2.56 mg Fremy’s salt equivalents (FSE/g f.w.)), as seen in Figure 3.

### 2.4. Assay of Antimicrobial Activity

Plants are important sources of potentially useful structures for the development of new chemotherapeutic agents. The first step towards this goal is the assessment of the *in vitro* antibacterial activity [17]. Several species of the *Lycium* genus or different plant parts of *L. barbarum* have been investigated related to their antibacterial or antifungal properties [6,18,19,20]. In this context, the antibacterial activity of *L. barbarum* flowers is worthy of investigation.

The results of testing the *L.barbarum* flowers extract for antimicrobial activities against both Gram-positive and Gram-negative bacteria are summarized in Table 4.

**Table 4:** Antibacterial activity and minimal inhibitory concentration of *L. barbarum* flower extract.

Bacterial Strains	Standard Antibiotic		Inhibition Zone (mm)	MIC (µg/mL)
	Gentamicin	Ciprofloxacin	*L. barbarum* Flowers	
*Staphylococcus aureus*	5.1 ± 0.2	5.2 ± 0.3	3.5 ± 0.1	75
*Bacillus subtilis*	4.2 ± 0.3	4.4 ± 0.1	2.7 ± 0.2	100
*Listeria monocytogenes*	7.3 ± 0.3	6.2 ± 0.2	1 ± 0.4	100
*Escherichia coli*	4.1 ± 0.2	5.1 ± 0.4	0	>100
*Salmonella typhimurium*	4.2 ± 0.1	4.3 ± 0.1	1 ± 0.1	100

Note: Each value is the mean ± SD of three independent measurements.

Results obtained in the present study revealed that *L. barbarum* flower extract was found to be more active on the Gram-positive bacterial strains; the best antibacterial activity was shown against *Staphylococcus aureus*. Previous investigated antibacterial activity of *L. barbarum* leaves extract showed that the best antibacterial effect was obtained on *Bacillus subtilis* [6].

## 3. Experimental Section

### 3.1. Plant Collection and Sample Preparation

The plant material with flowers of *L. barbarum* L. (Voucher No. 3574) was collected from Romanian spontaneous flora, in the summer of 2014, in Turda, Cluj County, Romania. A voucher specimen was deposited in the Department of Pharmaceutical Botany Herbarium of the Faculty of Pharmacy, “Iuliu Hatieganu” University of Medicine and Pharmacy, Cluj-Napoca, Romania. The fresh flower material was stored at −20 °C until the moment of analysis. One gram was weighed, grinded and extracted with 10 mL of 70% ethanol, two times for 30 min in an ultrasonic bath at room temperature. The samples were then centrifuged at 4500 rpm for 15 min, and the supernatant was recovered, filtered through a 0.45-μm micropore membrane (PTFE, Waters, WA, USA) and subjected to further analysis. In order to obtain more accurate data on flavonoid glycosides and aglycones concentration, each sample was analyzed before and after acid hydrolysis. Extractive solution (2 mL) was treated with 2 M hydrochloric acid (2 mL) and ascorbic acid solution (0.2 mL, 100 mg/mL), and the mixtures were heated at 80 °C on a water bath for 30 min, ultrasonicated for 15 min and heated for another 30 min at 80 °C. During the heating, methanol (1 mL) was added to the extraction mixture every 10 min, in order to ensure the permanent presence of methanol. The mixtures were centrifuged at 4000 rpm, and the solutions were diluted with distilled water in a 10-mL volumetric flask and filtered through a 0.45-μm filter before injection [8,11].

### 3.2. Chemicals

Chlorogenic acid, *p*-coumaric acid, caffeic acid, rutin, apigenin, quercetin, isoquercitrin, quercitrin, hyperoside, kaempferol, myricetol and fisetin were from Sigma (St. Louis, MO, USA); ferulic acid, sinapic acid, gentisic acid, gallic acid, patuletin and luteolin were from Roth (Karlsruhe, Germany); cichoric acid and caftaric acid were from Dalton (Toronto, ON, Canada). HPLC-grade methanol, ethanol, analytical grade orthophosphoric acid, hydrochloric acid and Folin–Ciocâlteu reagent were purchased from Merck (Darmstadt, Germany), hydrogen peroxide; ABTS (2,2′-azinobis-3-ethylbenzotiazoline-6-sulphonic acid), Fremy’s salt, sodium molybdate dihydrate, sodium nitrite, sodium hydroxide, sodium carbonate, sodium acetate trihydrate and anhydrous aluminum chloride were from Sigma-Aldrich (Steinheim, Germany). Trolox (6-hydroxy-2,5,7,8-tetramethylchroman-2-carboxylic acid) was obtained from Alfa-Aesar (Karlsruhe, Germany); HRP (horseradish peroxidase) was purchased from Sigma-Aldrich, Germany. Liposomes were obtained by suspending 5 mg/mL soybean lecithin (Alfa Aesar, Karlsruhe, Germany) in phosphate buffer followed by sonication and horse heart purified cytochrome *c* from Sigma-Aldrich (Steinheim, Germany). All spectrophotometric data was acquired using a Jasco V-530 UV-VIS spectrophotometer (Jasco International Co., Ltd., Tokyo, Japan).

### 3.3. HPLC/MS Analysis

The phenolic compounds were analyzed via an Agilent 1100 HPLC Series system (Agilent, Santa Clara, CA, USA) equipped with degasser G1322A, quaternary gradient pump G1311A and auto sampler G1313A. The separation was performed using a Zorbax SB-C18 reverse-phase column (100 mm × 3.0 mm i.d., 3.5-μm particle). The working temperature was 48 °C, and the detection of the compounds was performed at 330 nm (first 17 min from the chromatogram) and 370 nm (from 17 to 38 min) using a G1311A diode array detector system. Chromatographic data were processed using ChemStation software from Agilent. The mass spectrometer was an Agilent 1100 SL Series ion trap equipped with a turbo-ion spray (ESI, electrospray ionization) interface and was operated in negative ion mode. The parameters of the source were: temperature, 360 °C; dry gas, nitrogen; nebulizer, nitrogen at 65 psi. The mobile phase consisted of a binary gradient prepared from methanol and acetic acid solution 0.1% (*v*/*v*) in water. The gradient elution was: 0 to 35 min, from 5% to 42% methanol; isocratic elution followed, for the next 3 min, with 42% methanol. The flow rate was 1 mL/min; the injection volume was 5 μL; and data were collected at 330 nm. The identification of the polyphenols in the samples was made by comparison of their retention times, UV and MS spectra obtained with those of pure standards in the same chromatographic conditions and confirmed by HPLC/MS (Table 5). Quantitative determinations were made by using the external standard method. All compounds were identified by both standard addition and comparison of their retention times and MS spectra with those of standards [10,13].

**Table 5:** Retention times (R_T_) of standard polyphenolic compounds (min).

Peak No.	Phenolic Compound	*m*/*z*	R_T_ ± SD	Peak No.	Phenolic Compound	*m*/*z*	R_T_ ± SD
1	Caftaric acid	311	3.54 ± 0.05	10	Rutin	609	20.76 ± 0.15
2	Gentisic acid	153	3.69 ± 0.03	11	Myricetin	317	21.13 ± 0.12
3	Caffeic acid	179	6.52 ± 0.04	12	Fisetin	285	22.91 ± 0.15
4	Chlorogenic acid	353	6.43 ± 0.05	13	Quercitrin	447	23.64 ± 0.13
5	*p*-Coumaric acid	163	9.48 ± 0.08	14	Quercetin	301	27.55 ± 0.15
6	Ferulic acid	193	12.8 ± 0.10	15	Patuletin	331	29.41 ± 0.12
7	Sinapic acid	223	15.00 ± 0.10	16	Luteolin	285	29.64 ± 0.19
8	Hyperoside	463	19.32 ± 0.12	17	Kaempferol	285	32.48 ± 0.17
9	Isoquercitrin	463	20.29 ± 0.10	18	Apigenin	279	33.10 ± 0.15

Note: SD, standard deviation.

### 3.4. Determination of Phenolic Compounds

#### 3.4.1. Determination of Total Phenolic Content

Total phenolic content was determined with Folin–Ciocâlteu reagent according to the method described by Tămaș *et al.*, 2009, with some modifications. Two milliliters from each ethanolic extract were diluted 25 times and then mixed with Folin–Ciocâlteu reagent (1 mL) and distilled water (10.0 mL) and diluted to 25.0 mL with a 290-g/L solution of sodium carbonate. The samples were incubated in the dark for 30 min. The absorbance was measured at 760 nm, using a Jasco UV-VIS spectrophotometer. The standard curve was prepared by using different concentrations of gallic acid, and the absorbances were measured at 760 nm. TPC values were determined using an equation obtained from the calibration curve of the gallic acid graph (*R*^2^ = 0.999). Total polyphenolic content was expressed as mg gallic acid/g fresh plant material (mg GAE/g plant material) [18].

#### 3.4.2. Determination of Flavonoids Content

The total flavonoids content was calculated and expressed as quercetin equivalents after the method described in the Romanian Pharmacopoeia (Xth Edition) for *Cynarae folium*. Each extract (5 mL) was mixed with sodium acetate (5.0 mL, 100 g/L), aluminum chloride (3.0 mL, 25 g/L) and made up to 25 mL in a calibrated flask with methanol. Each solution was compared to the same mixture without reagent. The absorbance was measured at 430 nm. The total flavonoids content values were determined using an equation obtained from calibration curve of the quercetin graph (*R*^2^ = 0.999) [21].

### 3.5. Free Radical Scavenging Activity 

#### 3.5.1. TEAC Assay

The effects of the extract on the synthetic ABTS radical were estimated by the method previously described by Toma *et al.*, 2015, with some modifications. In the Trolox equivalent antioxidant capacity (TEAC) assay, the antioxidant capacity is reflected in the ability of the natural extracts to decrease the color, reacting directly with the ABTS cation radical. The latter was obtained by oxidation of 2,2′-azinobis(3-ethylbenzothiazoline-6-sulfonic acid (ABTS) with potassium persulfate. Original extracts were diluted 5 times, and 3 µL from the diluted extract were added to 997 µL ABTS solution. The amount of ABTS radical consumed by the tested compound was measured at 734 nm, after 30 min of reaction time. The evaluation of the antioxidant capacity was obtained using the total change in absorbance at this wavelength; all determinations being made in triplicate [22].

#### 3.5.2. Hemoglobin/Ascorbate Peroxidase Activity Inhibition Assay

The inhibition of hemoglobin ascorbate peroxidase activity assay (HAPX) was conducted according to the procedure previously described by Moț *et al.*, 2014. The reaction was triggered by the addition of methemoglobin (6 µM) to a mixture of ascorbate (160 µM), peroxide (700 µM) and extracts (5 µM) from the stock diluted 5 times, and it was monitored at 405 nm. This method allows us to evaluate the inhibition of ferryl formation by ascorbate in the presence of the tested compounds. An increase in the time of inhibition reflects the antioxidant capacity of the compound, whereas a decrease a prooxidant effect [23].

#### 3.5.3. Inhibition of Lipid Peroxidation Catalyzed by Cytochrome *c*

Liposomes were obtained by suspending 5 mg/mL soybean lecithin in phosphate buffer (20 mM, pH 7), followed by sonication for 15 min in an ultrasonic bath (using a Power Sonic 410 device). The liposome oxidation experiment was performed at room temperature, for 700 min, in the presence of cytochrome *c* (2 µM) and extracts (5 µL from the diluted extract) by monitoring the absorbance at 235 nm (the wavelength specific for liposome oxidation). This process monitors the formation of lipid conjugated dienes at the specified wavelength [6,15].

#### 3.5.4. EPR Spectroscopy Measurements

EPR measurements were carried out by the method previously described by Mocan *et al.*, 2014b, with some modifications. Appropriate extract dilutions (1:20) were prepared, and 25-μL aliquots were allowed to react for 60 min with an equal volume of a solution of Fremy’s salt (1 mM in phosphate buffer, pH 7.4). EPR spectra of Fremy’s radical were obtained with a Bruker Elexsys E500 spectrometer (Bruker, Billerica, MA, USA). The antioxidant activity expressed as mM Fremy’s salt reduced by 25 μL diluted extract was calculated by comparison to a control reaction with 25 μL Fremy’s salt 1 mM and 25 μL of extraction solvent [15].

### 3.6. Antimicrobial Activity Test

The antimicrobial activity testing was conducted by the methods that were previously described by our research group [6,12,15].

#### 3.6.1. Antimicrobial Activity Assay

The antimicrobial activity of the *L. barbarum* flower extract was evaluated by means of the agar well diffusion assay with some modifications. Fifteen milliliters of the molten agar (45 °C) were poured into sterile Petri dishes (Ø 90 mm). Cell suspensions were prepared, and 100 µL were evenly spread onto the surface of the agar plates of Mueller–Hinton agar (Oxoid, Basingstoke, U.K.). Once the plates had been aseptically dried, 6-mm wells were punched into the agar with a sterile Pasteur pipette. The different extracts (10 mg/mL) were dissolved in dimethylsulfoxide/water (1/9); 80 µL were placed into the wells, and the plates were incubated at 37 °C for 24 h. Gentamicin (25 µL/wells at a concentration of 4 µg/mL) and ciprofloxacin (5 µg/mL) were used as a positive control for bacteria. Antimicrobial activity was evaluated by measuring the diameter of circular inhibition zones around the well. Tests were performed in triplicate, and values are the averages of three replicates [6,12,15].

#### 3.6.2. Minimum Inhibitory Concentration

Based on the previous screening, the minimum inhibitory concentration (MIC) of *L. barbarum* flower extract was analyzed through the agar-well diffusion method. A bacterial suspension (10^5^ to 10^6^ CFU/mL) of each tested microorganism was spread on the nutrient agar plate. The wells (6 mm diameter) were cut from agar, and 60 µL of the extract dissolved in dimethyl sulfoxide (DMSO) at different concentrations (10, 20, 25, 50 75 and 100 µg/mL) were delivered into them. The plates were incubated at 37 °C for 24 h under aerobic conditions followed by the measurement of the diameter of the inhibition zone expressed in millimeters. The MIC was taken from the concentration of the lowest dosed well visually showing no growth after 24 h [6,12,15].

### 3.7. Statistical Analysis

The average of multiple measurements (triplicates or more) are listed in the tables together with the standard deviations. Statistical analysis was performed using the Excel software package.

## 4. Conclusions

To the best of our knowledge, this is the first report that reveals the polyphenolic analysis, antioxidant and antimicrobial activities of *L. barbarum* flowers ethanolic extract. The HPLC/MS analysis led to the identification and quantification of chlorogenic, *p*-coumaric and ferulic acids, isoquercitrin, rutin and quercitrin. The antioxidant potential of the extract was further assessed by several enzymatic or electron-transfer methods, indicating a correlation between the total phenolic and flavonoids content and the antioxidant effect. The antimicrobial activity was moderate against Gram-positive bacteria and lacking against *Escherichia coli*. These findings complete the reduced existing data and offer new perspectives for further pharmaceutical valorization of *L. barbarum* flowers.
